# How do members of the public feel about novel ecosystem interventions? A longitudinal study of emotional responses to restoration and adaptation in the Great Barrier Reef

**DOI:** 10.1007/s13280-025-02329-z

**Published:** 2026-01-09

**Authors:** Rana Dadpour, Stewart Lockie, Gillian Paxton, Brent W. Ritchie

**Affiliations:** 1 James Cook University Nguma-Bada Campus, 1/14-88 McGregor Rd, Smithfield, QLD 4878 Australia; 2 James Cook University Nguma-Bada Campus, 1/14-88 McGregor Rd, Smithfield, QLD 4878 Australia; 3 James Cook University Nguma-Bada Campus, 1/14-88 McGregor Rd, Smithfield, QLD 4878 Australia; 4 Business School, University of Queensland, Brisbane, QLD 4072 Australia

**Keywords:** Assisted ecosystem adaptation, Climate adaptation, Ecosystem restoration, Emotion, Great Barrier Reef, Reef restoration

## Abstract

Climate-responsive ecosystem interventions are increasingly proposed to support coral reef futures, yet the emotional dimensions of public responses remain poorly understood. Drawing on three large-scale surveys of Australians conducted in 2018, 2022, and 2024 (*n* = 8460), this study examines longitudinal shifts in emotional responses to six novel restoration and adaptation strategies in the Great Barrier Reef. Across all waves, positive emotions, especially hope and confidence, remained relatively stable, while several negative emotions fluctuated over time, with renewed intensification in 2024. Demographic position was associated with emotional variation, with younger and socioeconomically vulnerable respondents expressing stronger negative responses. We argue emotions are relational forces shaped by social conditions and perceived agency rather than purely individual dispositions. The study offers a transferable empirical framework for examining emotional responses to emerging ecological interventions, highlighting the value of integrating affective insight into adaptive environmental governance and public engagement in contexts of climatic uncertainty.

**Supplementary Information:**

The online version contains supplementary material available at 10.1007/s13280-025-02329-z.

## Introduction

Increasingly frequent mass coral-bleaching events and the forecast loss of tropical coral reefs worldwide under extreme climate change scenarios have prompted growing investment in the development of novel scientific and technological interventions aimed at helping reefs survive, recover, and adapt. The effectiveness of such interventions will depend on many things, including their acceptability to Indigenous peoples, local communities and stakeholders, and members of the broader public. As efforts to intervene in reef decline intensify, understanding the social dimensions of these responses has become critical to responsible innovation.

Nowhere is this challenge more visible than in the Great Barrier Reef (hereafter the GBR or ‘the Reef’), which, like many other coral reef ecosystems, is experiencing more frequent and prolonged mass bleaching events, with the fourth global bleaching event recorded in 2024 across the tropics, including Florida, the Red Sea, and the South Pacific (Klein et al. 2024). Modelling suggests that, in the absence of adaptation or acclimation, global warming of 2 °C will result in the collapse of critical reef functions and disappearance of nearly 40% of the GBR’s warm-water corals by 2050 (Quigley and Baird 2024). The social stakes are also considerable. Coral reefs sustain livelihoods, protect coastlines, and anchor cultural identities and psychological well-being for hundreds of millions of people globally (Anthony et al. 2017; Robinson et al. 2023; Bartelet et al. 2024).

In response, ambitious reef restoration and adaptation programmes have emerged, including Australia’s Reef Restoration and Adaptation Program (RRAP) and the KAUST Coral Restoration Initiative in Saudi Arabia. These initiatives are developing and trialling a range of interventions—categorised broadly as protective, restorative, and adaptive—with the aim of bolstering reef resilience under future climate scenarios (Bay et al. 2019; McLeod et al. 2022). Techniques such as marine cloud brightening and low-level fogging attempt to cool surface waters during heatwaves (Butcherine et al. 2023; Harrison 2024). Restorative efforts focus on enhancing coral recruitment and survival through engineered substrates, larval seeding, and scaled aquaculture (Randall et al. 2021; Kenyon et al. 2023). Adaptive strategies, meanwhile, aim to accelerate evolution by introducing heat-tolerant coral genotypes and symbionts or pre-conditioning juveniles for thermal stress (Scharfenstein et al. 2023; Selmoni et al. 2024).

These interventions are as social as they are technical and ecological. The risks, uncertainties, ethical contours and, ultimately, acceptability of intervention technologies are all interpreted through the lens of public perception (Anthony et al. 2020; Arning et al. 2020; Lockie et al. 2024; Baresi et al. 2025). Understanding the emotions that figure in public responses to prospective assisted adaptation interventions is particularly important. A growing body of research highlights the pivotal role emotions play in shaping environmental attitudes and behaviours (Smith and Leiserowitz 2013; Van der Linden 2014; Davidson 2018; Le et al. 2022; Myers et al. 2024; Varutti 2024) including reactions to climate challenges (Pihkala 2022). In a coastal community, for example, hope might enhance collective support for restorative actions while fear, in the absence of clear avenues for action, could lead to community disengagement (Curnock et al. 2019).

These emotional dynamics are particularly pronounced in the case of the GBR, where recognition of its outstanding universal value as a site of extraordinary beauty, biodiversity, evolutionary significance, and scale (as reflected in World Heritage designation) converges with its profound cultural, social and economic importance to Reef Traditional Owners and other Australians. Surveys show that more than three-quarters of Australians consider the Reef part of their national identity, and nearly 90% believe it is under threat from climate change (Goldberg et al. 2016). The degradation of coral reefs elicits intense emotional responses, not just because reefs are vibrant ecological systems, but because they are deeply woven into the identities, histories, and livelihoods of coastal communities and the wider public (Marshall et al. 2018; Cinner and David 2011). It is thus reasonable to expect that emotional responses to novel intervention technologies will have a major influence on attitudes towards their deployment.

Repeated bleaching events have elicited powerful emotions —grief, anger, anxiety, and longing—in what has been dubbed “Reef grief,” a collective mourning for an ecosystem in peril (Marshall et al. 2019). Around half of all residents, tourists, and tourism operators in the GBR region have reported significant Reef grief, reflecting sadness, despair, anger and even trauma as they witness Reef decline (Marshall et al. 2019). These emotions are not merely ephemeral feelings; they shape people’s sense of identity and responsibility. Many Australians feel a personal loss at the Reef’s degradation and a corresponding sense of duty to act to “protect what it means to be Australian” by saving the Reef (Goldberg et al. 2016; Marshall et al. 2019).

Emotions also influence the ways in which people imagine and articulate possible futures for the Reef. Paxton et al. (2024) show how public discourse about the GBR embodies a tension between narratives of inevitable loss and visions of alternative possibilities. While grief over the Reef’s decline fuels calls for urgent climate action, it also creates moral dilemmas around resilience-based management and technological interventions. Many community members grapple with whether to accept assisted ecosystem adaptation as a pragmatic option for helping imperilled reefs, to see ecological collapse as a potential catalyst for broader social and political change, or to hold both these possibilities together simultaneously (Paxton et al. 2024). These tensions reveal how emotions such as grief and hope are not just individual experiences (a theme we return to in the next section) but shared responses that influence Reef governance and responses to climate change more generally.

Emotions have long been central to the GBR story, galvanising public concern and political attention in ways that factual reports alone often could not. Media coverage of mass bleaching in 2016–2017 has been identified as a tipping point that made climate change feel more urgent to the public, as feelings of sadness, disgust, and anger towards the Reef’s destruction drove home the reality of the crisis (Curnock et al. 2019). As assisted adaptation strategies gain prominence, it becomes imperative to similarly examine how emotions influence both the possibilities and constraints of these interventions.

In the only major study of how emotions influence support for existing coral reef restoration projects in the GBR, Le et al. (2022) found the positive emotion of hope to be weakly associated with passive acceptance of restoration, while the negative emotion of guilt was moderately associated with active behavioural engagement. Most existing research, however, focuses on factors such as trust in institutions, scientific knowledge, and perceived risk (see, for example, Anthony et al. 2020; Lockie et al. 2024; Bartelet et al. 2025) with less attention paid to whether emotions such as hope, fear, worry, sadness, pride, or caution either motivate or hinder support (Wang et al. 2018; Le et al. 2022; Pihkala 2022). While a few studies have examined the role of emotions in shaping climate policy support (Smith and Leiserowitz 2014; Merk and Ponitzsch 2017; Myers et al. 2024), there remains limited understanding of how people feel about emerging reef interventions, how perceptions of these technologies shape emotional reactions, and how these responses develop over time.

The present study addresses this need through a longitudinal examination of emotional responses to proposed novel interventions in the Great Barrier Reef across three survey waves (2018, 2022, 2024). Drawing on large-scale surveys of Australian adults, we investigate how discrete emotions such as hope, caution, confidence, fear, and pride develop as people learn about and reflect on intervention options, and how demographic and social factors (e.g. age, gender, education, proximity to the Reef) shape these trajectories. By integrating these dimensions, we capture not only a momentary public mood, but the direction and momentum of emotional change over time, as well as the social contours of who feels what. We argue that emotional responses to interventions should not be treated as peripheral to rational policy and decision-making, but as fundamental to effective public engagement and intervention development. Accordingly, this study addresses three guiding questions: (1) How have discrete emotions towards novel reef interventions changed across 2018, 2022, and 2024? (2) How do demographic and social factors shape these emotional patterns over time? and (3) What implications do these temporal and social patterns hold for public engagement and the governance of reef restoration and adaptation?

## Theoretical framework

Emotions are pervasive in every domain of human life and decision-making, yet their specific role in shaping responses to pressing environmental challenges—such as coral reef decline and associated technological interventions—has only recently begun to garner scholarly attention (Davidson 2018; Le et al. 2022; Pihkala 2022). It is important to note, in this context, that the ways in which authors conceptualise emotion is variable. Some adopt a narrow definition of emotion as peoples’ conscious awareness of how they feel in relation to a particular object (and thus something to be distinguished from affects, or unconscious bodily feelings) while others use the term to connote a broader array of conscious and unconscious, subjective and embodied feelings (Pihkala 2022). We adopt this second, and looser, conceptualization in this study as it is closer to the everyday understanding of emotions with which participants in our research will have interpreted and responded to our questions. In doing so, however, we note the salience of theoretical work on emotion to the interpretation of our results.

Sociological theory, for example, stresses that emotions are not merely individual, internal states. While deeply personal, emotions emerge, circulate, and gain momentum through social interactions and shared narratives (Parkinson 1996; Boiger and Mesquita 2012). Emotional responses to ecosystem intervention are shaped by public discourses and collective visions for the future (Smith and Leiserowitz 2014; Van der Linden 2014) along with gendered emotional norms (Fischer 2000), age-related affective shifts (Carstensen et al. 2011), the influence of task-oriented groups (Kelly and Barsade 2001), and so on. The question of how people feel about prospective intervention in the GBR is thus a question that speaks to the multitude of ways in which coral ecosystems, scientific interventions, media images, government policies, social structures, and cultural narratives interact to co-produce collective emotions which, in turn, influence public support or resistance to intervention measures. It is a question also that challenges the strict separation of emotion from cognition in public perceptions of risk (Lupton 2013).

One key driver of emotional reactions is the perception that one’s “objects of care”—places, people, or cultural symbols that hold personal and communal significance—are threatened by climate change (Wang et al. 2018). This sense of threat can fuel both supportive responses such as a willingness to back restoration initiatives, and critical or wary stances, including caution or outright opposition (Davidson 2018; Bartelet et al. 2025). From this vantage, emotions serve dual functions: they can animate social ties and catalyse collective action, but they can also reflect underlying anxieties and uncertainties about technological interventions (Le et al. 2022; Myers et al. 2024). As Ahmed (2004) notes, the “stickiness” of emotions to particular objects, like coral reefs, imbues them with moral weight and shapes how public conversations unfold, thereby influencing not only personal risk perceptions but also broader policy debates.

Emotions such as hope, anger, and pride are integral to the formation of social capital in environmental contexts. By creating or reinforcing group bonds, emotional expressions can become powerful motivators for joint initiatives, advocacy, and public engagement (Sarı et al. 2024). Within reef governance, shared hope may inspire support for restoration and adaptation technologies, while collective anger or sadness might channel criticism towards perceived institutional inaction (Curnock et al. 2019). The ability of emotions to galvanise or fragment social networks underlines their significance as part of a region’s social capital, in that they influence cooperation, trust, and a sense of mutual obligation (Boiger and Mesquita 2012).

Moreover, emotions connect individuals and communities in ways that build resilience and stimulate collective problem-solving. When harnessed constructively, strong feelings like pride or optimism can help maintain momentum for interventions over the long term, especially when ecological outcomes are slow to materialise or remain uncertain (Smith and Leiserowitz 2014; Bartelet et al. 2025). In this way, the Reef’s emotional capital can function as a resource, just as valuable as financial, institutional, or ecological resources, by shaping how communities navigate scientific innovation and respond to socio-ecological threats (Marshall et al. 2019; Le et al. 2022).

In empowering individuals to address environmental challenges, emotional awareness—the ability to recognise and understand one’s own emotions and those of others—proves fundamental (Williamson and Thulin 2022). Environmental agency often hinges on whether people feel both capable and motivated to act effectively in the face of ecological threats (Williamson and Thulin 2022). In this regard, emotions lay the groundwork: fear or worry can spur urgent protective measures, while hope and pride can bolster perseverance and continued advocacy (Wang et al. 2018). Consequently, cultivating emotional awareness becomes essential for fostering environmental engagement (Pihkala 2022). When policymakers and practitioners acknowledge, rather than dismiss, the depth of public feeling about the Reef, they create opportunities for more inclusive and participatory decision-making (Cinner and David 2011).

In particular, scholars highlight those negative emotions—such as sadness, fear, or caution—are not simply impediments to acceptance of novel interventions; when met with credible information, transparent communication, and avenues for tangible involvement, these emotions can be channelled into constructive civic engagement (Lupton 2013; Waters et al. 2024). Conversely, unaddressed or dismissed emotions risk leading to cynicism or withdrawal from public processes (Nightingale et al. 2022). Positive emotions like hope and confidence, on the other hand, have been shown to encourage an ongoing commitment to change, although they can wane without visible progress or social recognition (Smith and Leiserowitz 2014; Williamson and Thulin 2022).

Taken together, these perspectives underscore that an emotionally attuned approach to reef governance is vital. Emotions operate as collective and relational forces, shaping social capital, fueling or tempering environmental agency, and guiding how communities process uncertainties surrounding large-scale interventions. By engaging with the emotional landscape rather than attempting to bypass it, those involved in designing, implementing, or communicating reef interventions can better align their efforts with the nuanced realities of public sentiment. This alignment does not guarantee unanimous support for novel technologies; rather, it offers a grounded basis for dialogue and cooperation, particularly in contexts as ecologically significant and symbolically charged as the GBR.

## Materials and methods

This manuscript draws on data collected as part of a broader inquiry into public perceptions of current and emerging management interventions for the Great Barrier Reef, conducted within the framework of Australia’s Reef Restoration and Adaptation Program (RRAP). A collaborative initiative between the Australian Government’s Reef Trust and the Great Barrier Reef Foundation, RRAP’s objective is to equip Reef managers with a scientifically validated, ecologically sustainable, socially acceptable, technically feasible, and economically viable portfolio of interventions designed to enhance the Reef’s resilience in the face of climate change.

Data were collected through three large-scale national surveys conducted in 2018, 2022, and 2024. Respondents were drawn from two key sampling frames: a national sample, representative of the broader Australian population, and a 50GBR sample, comprising individuals residing within 50 km of the GBR coastline. In each survey wave, data were gathered via online surveys administered by a market research company using online panels. Quota sampling ensured representativeness, with national quotas calibrated against Australian census data (incorporating gender, age, and geographic distribution across urban and rural areas), while the resident sample was guided by soft quotas aligned with Queensland’s demographic composition. To ensure data integrity, incomplete responses and surveys completed at an implausibly rapid pace were excluded.

Each survey was designed to capture a broad range of demographic indicators including age, education, employment status, and Aboriginal and Torres Strait Islander identity, alongside public attitudes towards an expanding portfolio of reef intervention technologies and associated emotional responses. A combined sample of *n* = 8460 Australian adults were first presented with a detailed description of one of six novel intervention strategies, outlining its purpose, mechanisms, and potential implications (see Appendix A for comprehensive descriptions in Supplementary S1).

Following this, they were asked to reflect on their emotional response to the intervention by responding to the prompt: “How do you feel when you consider the type of approach/technology outlined earlier?” Emotional reactions were measured using a Likert-type scale ranging from 1 (“not at all”) to 7 (“extremely”), capturing the intensity of ten distinct emotions: Cautious, Worried, Sad, Powerless, Scared, Proud, Hopeful, Happy, Confident, and Relieved. These emotions were developed based on public opinion studies related to geoengineering and climate change mitigation (Midden and Huijts 2009; Braun et al. 2017; Merk and Ponitzsch 2017; Carr and Yung 2018). This approach allowed for a nuanced assessment of both negative and positive emotional responses, providing insights into the emotional landscape surrounding public perceptions of the intervention.

To aid interpretation of temporal patterns, we note that each survey wave occurred against distinct external contexts that may shape the salience of emotions. The 2018 wave followed the 2016–2017 mass bleaching events on the GBR, when public awareness of reef decline was high. The 2022 wave was conducted in the aftermath of COVID-19, a period marked by shifting public attention and recovery dynamics. The 2024 wave coincided with the fourth global bleaching event, widely reported across tropical reefs. While our design does not identify causal effects of these contexts, their coincidence with the survey fieldwork may have influenced baseline emotional tone and we interpret year-to-year differences with this contextual backdrop in mind.

### Statistical analyses

To understand emotional responses towards novel interventions in the GBR, we conducted a series analysis to assess the distribution of emotions, explore interrelationships among them, evaluate demographic differences, and examine temporal trends. Descriptive statistics were first computed to summarise the central tendency and dispersion of each emotion. Means and standard deviations provided an overview of emotional prevalence, while skewness and kurtosis values, along with their associated z-scores, were assessed to determine the distributional properties of the data. Pearson’s correlation coefficients were then used to explore relationships among the ten discrete emotions, revealing distinct clustering patterns. Hierarchical clustering analysis using Ward’s method further refined these insights by empirically identifying emotional groupings based on proximity scores.

To assess demographic variations in emotional responses, we employed a Multivariate Analysis of Variance (MANOVA), incorporating key demographic variables such as gender, age, education, employment status, and proximity to the GBR. Four multivariate test statistics—Pillai’s Trace, Wilks’ Lambda, Hotelling’s Trace, and Roy’s Largest Root—were examined to determine the significance of demographic effects. Where MANOVA results indicated significant differences, follow-up univariate analyses (ANOVA) were conducted to examine specific effects on individual emotions. Effect sizes were reported using Partial Eta Squared (η^2^) to provide an indication of the magnitude of demographic influences.

To evaluate temporal shifts in emotional responses, a longitudinal MANOVA was conducted across three survey waves (2018, 2022, 2024). Follow-up ANOVAs were performed to assess year-to-year differences in individual emotions, with post hoc comparisons (Tukey HSD and Bonferroni) identifying specific changes over time. A Kruskal–Wallis H test was also conducted as a nonparametric alternative to confirm the robustness of the parametric results.

Finally, to examine whether different categories of reef interventions elicited distinct emotional reactions, we conducted a separate MANOVA comparing emotional responses across protective (e.g. cloud brightening), restorative (e.g. coral seeding), and adaptive (e.g. genetic interventions) technologies. Effect sizes were reported using Partial Eta Squared (η^2^). Together, these analyses provide a comprehensive assessment of the distribution, structure, and evolution of emotional responses to novel reef interventions, capturing demographic and temporal patterns while contextualising the emotional landscape of public engagement.

## Results

### Descriptive statistics of emotional responses (Appendix B in Supplementary S1)

**Fig. 1:**
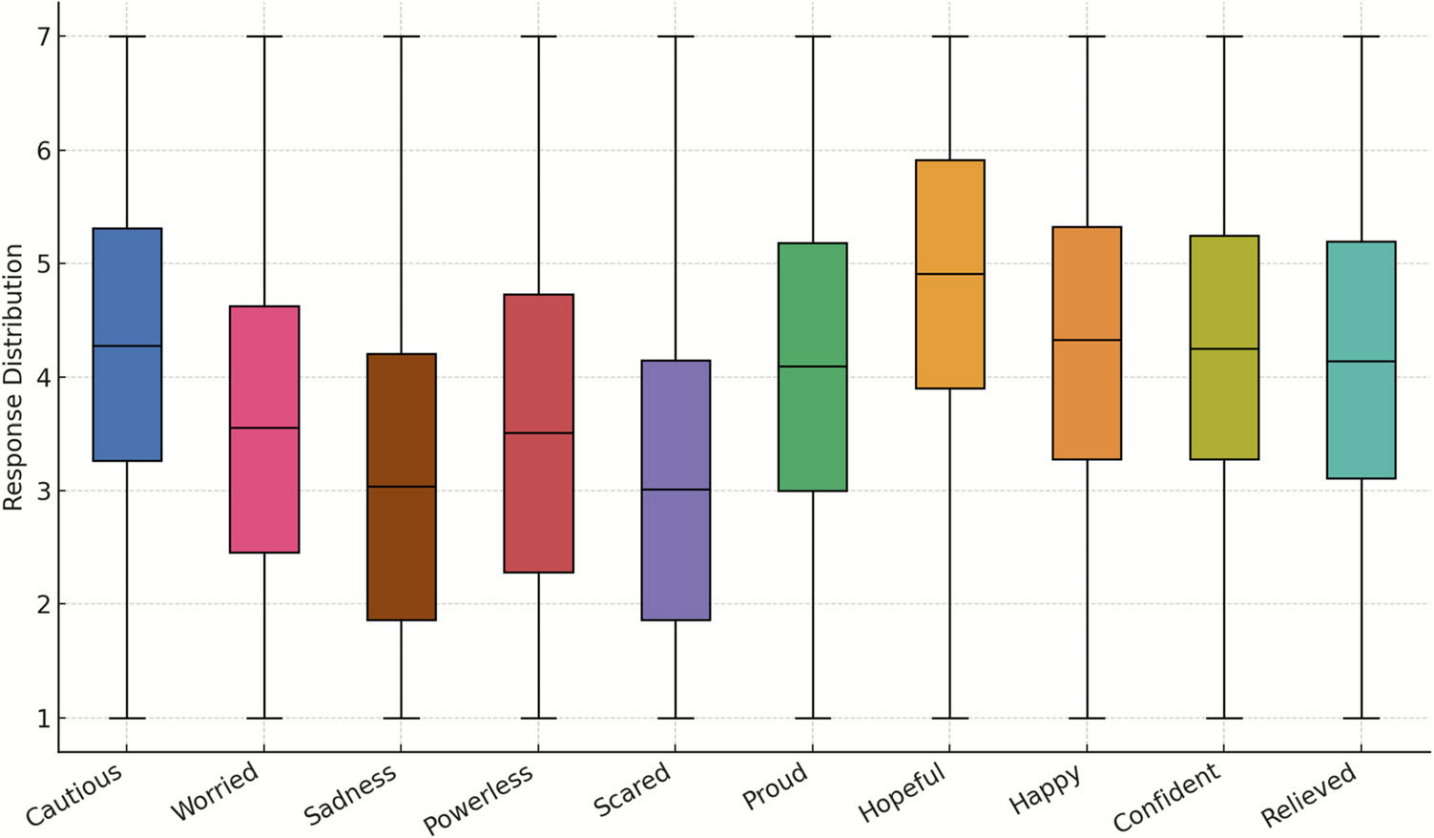
Distribution of emotional responses to novel interventions in the Great Barrier Reef

The distribution of emotional responses among respondents revealed a higher prevalence of positive than negative emotions (Fig. 1). Hopeful was highest (*M* = 4.90, SD = 1.46), indicating a widely shared constructive outlook on reef futures. On the negative side, Cautious was most salient (*M* = 4.29), exceeding Worried and Scared (Ms = 3.02- 3.53), consistent with risk awareness without widespread distress. The spread of responses was moderate (SDs = 1.46–1.75); negative emotions (e.g. Worried, Sad, Powerless) were more variable than positives, implying greater heterogeneity in concern.

Distributions for high-mean positives were left-skewed: Hopeful (skew = − 0.544) and Confident (− 0.250), meaning more respondents clustered at higher levels. In contrast, key negatives were right-skewed: Sad (0.490) and Scared (0.479), indicating relatively few highly distressed respondents and many reporting low intensity. Cautious sat near symmetry (− 0.125), suggesting a broadly shared, balanced caution.

Hopeful showed slight positive kurtosis (0.189), i.e. mild clustering near the mean with occasional extremes. Sad (− 0.626) and Scared (− 0.563) were flatter (platykurtic), indicating more even dispersion across the scale rather than sharp peaks. Confident (− 0.071) and Cautious (− 0.109) were close to normal, meaning many respondents report at middling-to-high levels.

Several distributions moved towards greater symmetry: Cautious shifted from − 0.237 (*Z* = − 4.39, *p* < 0.05) to − 0.015 (*Z* = − 0.34, *p* > 0.05); Sad from 0.520 (*Z* = 9.63, *p* < 0.05) to 0.346 (*Z* = 7.86, *p* < 0.05); Powerless from 0.256 (*Z* = 4.74, *p* < 0.05) to 0.195 (*Z* = 4.43, *p* < 0.05). In contrast, Scared increased in positive skew from 0.399 (*Z* = 7.39, *p* < 0.05) to 0.507 (*Z* = 11.52, *p* < 0.05), and Worried rose modestly from 0.131 (*Z* = 2.43, *p* < 0.05) to 0.190 (*Z* = 4.32, *p* < 0.05). Positive emotions generally showed smaller negative skew by 2024 (e.g. Proud − 0.224 → − 0.101; Hopeful − 0.638 → − 0.433; similar for Happy, Confident). The overarching pattern is that several emotions became more balanced over time, while fear- and worry-related responses remained more lopsided.

Overall, and integrating the numbers, it is noticeable that high means for Hopeful (4.90) and Cautious (4.29), coupled with left-skew for positives (e.g. Hopeful − 0.544) and right-skew for negatives (e.g. Sad 0.490; Scared 0.479), show a public that is durably hopeful and broadly—now more uniformly—cautious, with acute distress concentrated in a minority.

### Clusters of emotions (Appendix C in Supplementary S1)

The Spearman’s correlation matrix identified two primary groups of emotions, highlighting distinct patterns of associations among positive and negative affective responses. The first group encompassed negative emotions: Cautious, Worried, Sadness, Powerless, and Scared, demonstrating strong intercorrelations. The strongest associations occurred between Sadness and Scared (*ρ* = 0.678) and Worried and Scared (*ρ* = 0.646), suggesting that fear was closely linked with sadness and worry. Moreover, Sadness, Worried, and Powerless formed a particularly coherent subcluster, indicating that these emotions frequently co-occurred, representing a generalised state of distress or apprehension.

The second group included positive emotions: Proud, Hopeful, Happy, Confident, and Relieved, all showing strong interconnections. The most substantial correlations were observed between Happy and Confident (*ρ* = 0.719), Happy and Relieved (*ρ* = 0.695), and Happy and Proud (*ρ* = 0.680). These correlations underscore the tightly interconnected nature of these positive emotional states, indicating that experiencing one positive emotion significantly relates to experiencing others within this group.

As anticipated, emotions across the positive and negative groups displayed negative correlations, although these associations remained modest in magnitude. For example, Worried negatively correlated with Happy (*ρ* = − 0.126), Confident (*ρ* = − 0.133), and Hopeful (*ρ* = − 0.121), suggesting a modest inverse relationship between worry and positive emotions. However, these relationships were relatively weak, indicating that the presence of negative emotions did not entirely preclude the simultaneous experience of positive emotions. Notably, Cautious exhibited comparatively moderate correlations within the negative group, most strongly associated with Worried (*ρ* = 0.478). This moderate correlation suggests that while caution may interact with worry, it reflects a more measured, less intensely negative emotional response.

**Fig. 2:**
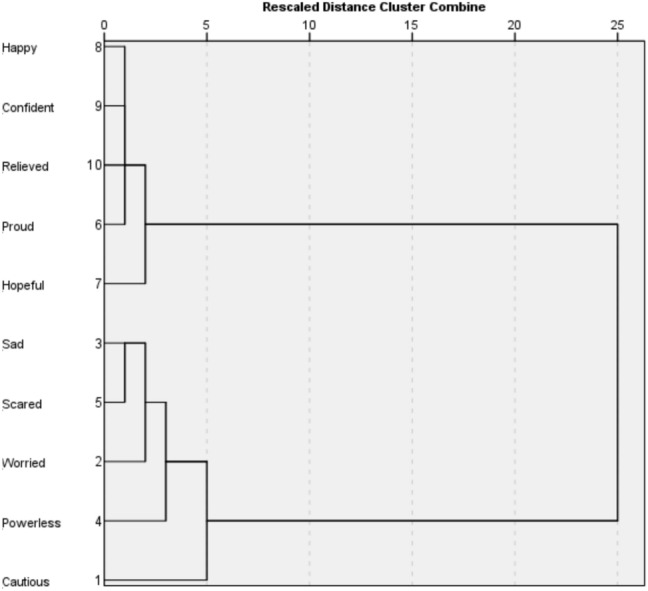
Hierarchical cluster dendrogram of emotional responses to reef novel interventions, generated using Ward’s method

To further refine the emotional structure underlying public responses, we conducted hierarchical clustering analysis using Ward’s method to empirically define emotion groupings. This method allows us to move beyond correlation-based relationships and identify discrete emotional profiles that shape perceptions of reef interventions. We identified two primary emotional clusters, with evidence suggesting a possible intermediate emotional space. The first cluster consisted of Worried, Scared, Sad, and Powerless, forming a concern-driven emotional category characterised by distress and uncertainty. Individuals who experienced one of these emotions were highly likely to experience others in the same group. The second cluster included Hopeful, Happy, Proud, and Confident, signifying an optimism-driven category, in which individuals who perceived reef interventions as realistic measures to improve ecological outcomes similarly expressed multiple reinforcing emotions (Fig. 2).

Analysis of emotional proximities revealed that Scared and Sadness were the most closely associated emotions (proximity = 16 432), indicating that distress in response to interventions was often characterised by overlapping feelings of fear and grief. Among positive emotions, Happy and Confident exhibited the strongest proximity (proximity = 9951), confirming that positive affective engagement was grounded in the co-occurrence of assurance and satisfaction. The largest emotional distance was observed between Sadness and Hopeful (proximity = 77 763), showing that individuals who felt sadness towards reef interventions were the least likely to express hopefulness, signalling a pronounced emotional divide between concern- and optimism-driven responses.

Cautious occupied an intermediate position, showing moderate proximity to both clusters. Its closest association was with Worried (proximity = 25 558), reflecting a measured and anticipatory stance more cognitive than affective in tone. Its larger distances from Sadness (43 022) and Scared (40 194) reinforce this interpretation of caution as an evaluative rather than distressed response. Relieved aligned primarily with the optimism-driven cluster but maintained a moderate proximity to Cautious (38 429), suggesting a transitional emotional state in which acceptance of interventions coexists with lingering apprehension.

### Demographic factors: multivariate findings

#### Gender

The effect of gender on emotions was significant (*p* < 0.001) across all four multivariate test statistics (Pillai's Trace, Wilks' Lambda, Hotelling’s Trace, and Roy’s Largest Root). The Partial Eta Squared (*η*^2^) was 0.006, indicating that gender accounted for only 0.6% of the variance in emotions. This suggests that while gender differences exist, they explain a very small proportion of the overall variation in emotional responses.

#### Proximity to GBR

Multivariate tests showed statistically significant differences in emotional responses between the National and 50 km GBR samples (Pillai’s Trace = 0.008, *p* < 0.001), but the effect size was extremely small (*η*^2^ ≤ 0.003), indicating that these differences are not practically meaningful. The National sample reported slightly higher Sadness (*p* < 0.001) and marginally higher Worry (*p* ≈ 0.05), whereas respondents within the 50 km GBR region expressed somewhat greater Pride, Happiness, and Confidence (all *p* < 0.05). Other emotions, including Hopefulness and Relief, did not differ significantly between groups.

#### Aboriginal and Torres Strait Islander identity

Aboriginal identity had a statistically significant but very small effect on emotions (*p* = 0.004, *η*^2^ = 0.002). Torres Strait Islander (TSI) identity also showed a significant effect (*p* < 0.001, *η*^2^ = 0.003–0.005), while the interaction between Aboriginal and TSI identity was significant as well (*p* < 0.001, *η*^2^ = 0.002–0.005). However, all effect sizes (*η*^2^ ≤ 0.005) were extremely small, indicating that although statistically significant, these differences are not practically meaningful.

#### Age

Younger individuals (18–39) reported stronger emotional responses overall, feeling more Worried, Scared, Sad, Proud, Happy, Confident, and Relieved compared to older respondents. Positive emotions such as Hopeful, Proud, Happy, and Confident declined slightly with age, particularly beyond 50 years, while negative emotions like worry and fear were also more pronounced among younger participants. Middle-aged respondents (40–49) showed the highest cautiousness (B = 0.155, p = 0.001). Older individuals (60+) reported lower emotional intensities across nearly all emotions. Although these differences were statistically significant, effect sizes were very small (*η*^2^ ≤ 0.015).

#### Education

Education had a statistically significant multivariate effect on emotional responses (*Pillai’s Trace* = 0.023, *F*(40, 33 772) = 4.978, *p* < 0.001, η^2^ = 0.006). Although the effect size was small, several consistent patterns emerged. Participants with lower education levels (those who did not complete Year 12) reported higher cautiousness (*B* = − 0.317, *p* < 0.001) and powerlessness (*B* = 0.231, *p* = 0.001) than those with postgraduate degrees. Respondents with higher education (undergraduate and postgraduate) expressed greater confidence (B = − 0.338, *p* < 0.001) and happiness (*B* = − 0.306, *p* < 0.001), while those with post-secondary qualifications showed more moderate emotional patterns between the two ends of the spectrum. Overall, education explained less than 1 per cent of the variance in emotional responses (*η*^2^ ≤ 0.007), indicating the influence was limited in magnitude.

#### Employment

Full-time and part-time employees reported significantly higher confidence (*B* = 0.503, *p* < 0.001, *η*^2^ = 0.019), happiness (*B* = 0.458, *p* < 0.001, *η*^2^ = 0.015), pride (*B* = 0.493, *p* < 0.001, *η*^2^ = 0.015), hopefulness (*B* = 0.398, *p* < 0.001, η^2^ = 0.005), and relief (*B* = 0.404, *p* < 0.001, *η*^2^ = 0.015) compared with unemployed respondents. Retired individuals reported feeling significantly less worried (*B* = − 0.213, *p* = 0.013) and scared (*B* = − 0.221, *p* = 0.013) than the unemployed, suggesting lower emotional distress. Students expressed moderate pride and hopefulness but also reported slightly higher powerlessness (B = − 0.258, *p* = 0.038). Carers and those in home duties showed balanced emotional patterns across both positive and negative emotions. Although statistically significant (Pillai’s Trace = 0.050, *F*(50, 42,215) = 8.457, *p* < 0.001, *η*^2^ = 0.010), employment status explained less than 2% of the variance in emotional responses (*η*^2^ ≤ 0.019).

#### Cross-demographic insights

By combining these demographic factors, we observe broad patterns. Younger individuals with lower education and unemployment experienced the most intense emotional responses, especially fear, sadness, and worry. Older, educated, and employed individuals exhibited more controlled emotional responses, with lower negative emotions and higher positive emotions. Employment appears to buffer against negative emotions more effectively than age or education alone. Education moderated emotions, especially reducing fear and sadness, but had a weaker effect on positive emotions. Retirement was linked to lower emotional intensity overall. Students showed higher hopefulness, likely reflecting optimism and openness to new technologies.

### Interventions’ technologies (block) and emotions

The multivariate tests confirmed a statistically significant but small effect of intervention type on emotional responses (Pillai’s Trace = 0.029, *p* < 0.001, *η*^2^ = 0.006). This suggests that while participants differentiated between interventions emotionally, these differences did not carry substantial weight in shaping overall emotional landscapes. These findings, however, highlight the complexity of public emotions in response to different technological approaches to reef intervention.

#### Protective technologies (fogging and cloud brightening)

Protective interventions evoked significantly lower negative emotions, particularly for Cautious (*B* = − 0.372 to − 0.292, *p* < 0.001), Worried (*B* = − 0.413 to − 0.346, *p* < 0.001), and Sadness (*B* = − 0.210 to − 0.312, *p* ≤ 0.002). Both also showed slight reductions in Powerlessness (*B* = − 0.137 to − 0.169, *p* ≤ 0.05). However, neither intervention meaningfully increased positive emotions such as Hopeful or Happy.

#### Restorative technologies (rubble stabilisation and coral seeding)

Restorative interventions were associated with lower negative emotions across Worried (*B* = − 0.470 to − 0.559, *p* < 0.001), Sadness (*B* = − 0.253 to − 0.328, *p* < 0.001), and Scared (*B* = − 0.337 to − 0.513, *p* < 0.001), while simultaneously showing modest positive shifts in Proud (*B* = 0.290 to 0.322, *p* < 0.001), Hopeful (*B* = 0.285 to 0.329, *p* < 0.001), Happy *B* = 0.335 to 0.355, *p* < 0.001), Confident (*B* = 0.309 to 0.371, *p* < 0.001), and Relieved (*B* = 0.331–0.365, *p* < 0.001).

#### Adaptive technologies (natural breeding and genetic engineering)

Adaptive interventions generated the most polarised reactions. Natural Breeding generated higher positive affect, notably greater Hopeful (*B* = 0.329, *p* < 0.001), Happy (*B* = 0.355, *p* < 0.001), Confident (*B* = 0.371, *p* < 0.001), and Relieved (*B* = 0.365, *p* < 0.001), and lower negative emotions, including Scared (*B* = − 0.513, *p* < 0.001) and Sadness (*B* = − 0.328, *p* < 0.001). In contrast, Genetic Engineering (the reference category) consistently elicited the highest levels of caution, worry, and fear, and the lowest positive emotions.

### Evolution of emotional responses to novel interventions over time

**Fig. 3:**
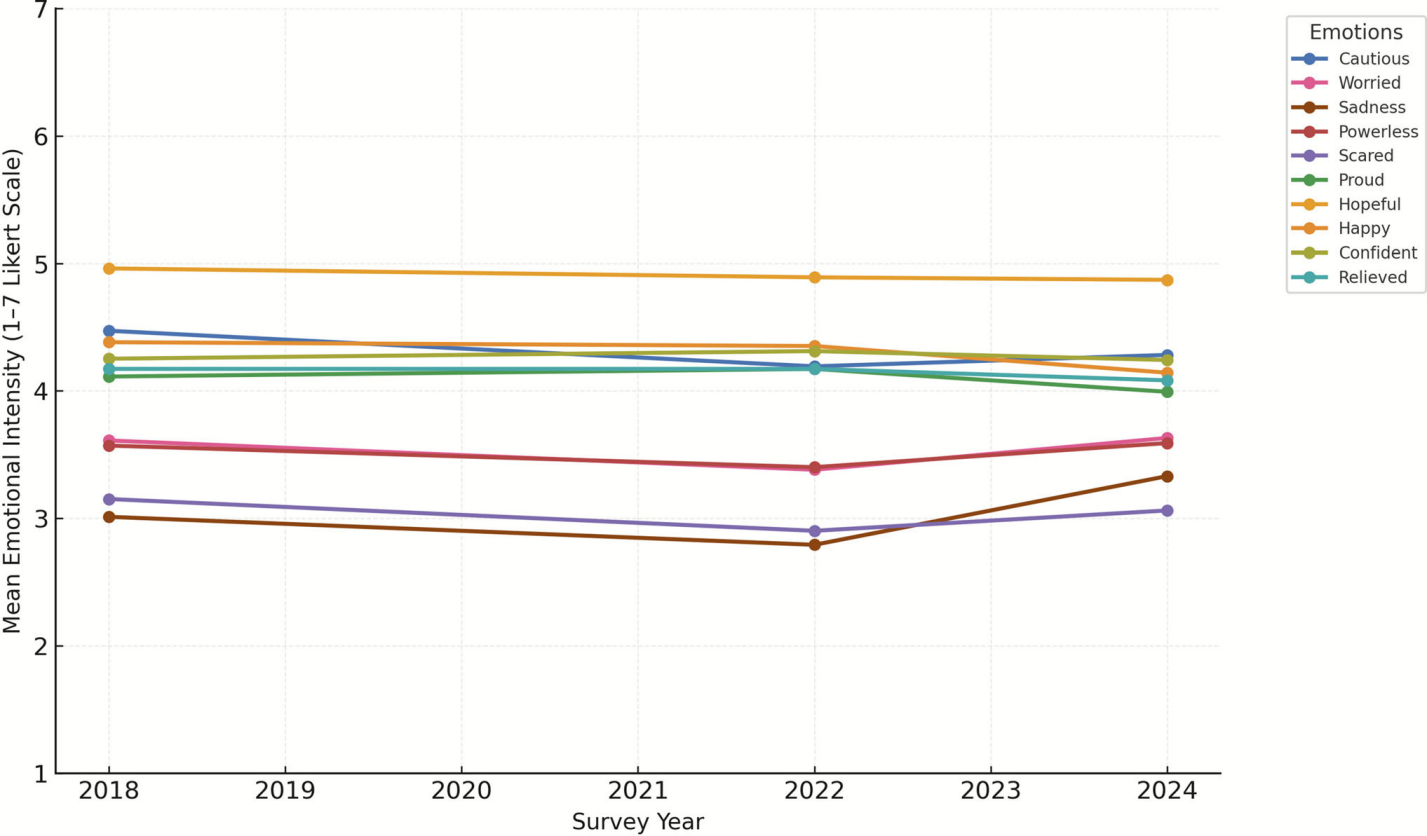
Temporal shifts in emotional responses to novel interventions in the Great Barrier Reef (2018–2024)

The results of the Multivariate Analysis of Variance (MANOVA) indicate that survey year had a statistically significant effect on emotional responses to reef intervention technologies (Pillai’s Trace = 0.042, *F*(20, 16,896) = 18.31, *p* < 0.001). All four multivariate test statistics (Pillai’s Trace, Wilks’ Lambda, Hotelling’s Trace, and Roy’s Largest Root) confirmed these differences. However, the Partial Eta Squared value (*η*^2^ = 0.021) suggests that the effect size is small, indicating that while differences existed, the magnitude of change across years was limited (Fig. 3).

We conducted Univariate Analyses of Variance (ANOVAs) to examine differences across survey years (2018, 2022, and 2024) for each emotion individually. Results indicated that survey year significantly affected eight of the ten emotions, with Hopeful (*F*(2,8456) = 2.533, *p* = 0.080, *η*^2^ = 0.001) and Confident (*F*(2,8456) = 2.470, *p* = 0.085, *η*^2^ = 0.001) showing no significant differences across years. Among the significant results, Sadness displayed the largest year effect (*F*(2,8456) = 78.176, *p* < 0.001, *η*^2^ = 0.018), followed by Worried (*F*(2,8456) = 23.680, *p* < 0.001, *η*^2^ = 0.006) and Happy(*F*(2,8456) = 21.130, *p* < 0.001, *η*^2^ = 0.005). All other significant effects were small (*η*^2^ ≤ 0.004), indicating modest temporal variations in emotional responses.

Post hoc analyses (Tukey HSD and Bonferroni) identified several significant year-to-year differences. Negative emotions generally decreased between 2018 and 2022, suggesting a temporary decline in emotional distress, but most increased again in 2024. Sadness displayed the largest shift, falling from 3.01 in 2018 to 2.79 in 2022 (*p* < 0.001) before rising sharply to 3.33 in 2024 (*p* < 0.001). Worried followed a similar pattern, decreasing from 3.61 in 2018 to 3.38 in 2022 (*p* < 0.001) and increasing to 3.63 in 2024 (*p* < 0.001). Cautious also declined between 2018 (*M* = 4.47) and 2022 (*M* = 4.19, *p* < 0.001) and then rose slightly to 4.28 in 2024 (*p* = 0.047). Powerless and Scared mirrored this general pattern, with small declines from 2018 to 2022 followed by minor increases in 2024 (*p* < 0.001 for both), indicating renewed apprehension in the most recent survey year.

Among positive emotions, Happy showed the most consistent decline over time, dropping from 4.38 in 2018 to 4.35 in 2022 and then to 4.14 in 2024 (*p* < 0.001), while Proud decreased modestly from 4.17 in 2022 to 3.99 in 2024 (*p* < 0.001). Relieved exhibited a small but statistically significant reduction (*F*(2,8456) = 3.469, *p* = 0.031), with slightly lower means in 2024 (*M* = 4.08) compared to earlier years. Hopeful which was the most consistently reported positive emotion, remained relatively stable from 4.96 in 2018 to 4.87 in 2024 (*p* = 0.080), and Confident also showed no significant year-to-year differences (*p* = 0.085).

To further validate our findings and address potential concerns about the ordinal nature of Likert-scale measures, we conducted a Kruskal–Wallis H test as a nonparametric alternative. The Kruskal–Wallis tests indicated significant year-to-year variation across all ten emotions (*p* < 0.05), with the strongest differences observed for sadness, worry, and caution. However, consistent with the MANOVA results (*η*^2^ ≤ 0.021), these effects were small in magnitude. These results reinforce the robustness of the parametric approach.

## Discussion

When asked how they felt about the prospect of novel technological interventions in the GBR, the strongest response was the expression of hope. Hope can be understood as a future-oriented, or anticipatory, state that helps people imagine better futures (Geiger et al. 2023). It has been identified as a cornerstone emotion for sustaining long-term commitment to climate action (Smith and Leiserowitz 2014; Le et al. 2022). It propels forward-thinking attitudes, fosters resilience in the face of discouraging environmental news, and can bind communities together in collective projects of conservation or restoration. Our findings suggest that the feeling of hope through exposure to prospects for intervention is widely experienced by members of the Australian public, albeit not always to the same extent, meaning a core majority experienced moderate hope while a smaller subset experienced extreme hope.

Both hope and confidence remained relatively stable across the survey years. Confidence gestures towards people’s sense that these interventions can be effective and are backed by credible and trustworthy science. The fact that neither metric showed a stark decline confirms that a baseline level of hope and confidence endured despite the fluctuations we observed in negative emotions and the slight downturn observed in happiness and pride.

Taken together, these results suggest that while respondents were aware of challenges faced by the Reef, and distressed by reports of decline in Reef condition, information about potential interventions provided a cognitive and emotional resource with which to build hope and, to a lesser extent, confidence, in better ecological outcomes. This highlights the need to communicate both the successes of restoration and adaptation efforts, and the limitations or risks involved.

The decline in negative emotions between the initial (2018) and intermediate (2022) survey points was consistent with prior research demonstrating that, as the public becomes more informed about cutting-edge interventions, initial fears or misconceptions can subside (Curnock et al. 2024; Hasley et al. 2023). In part, this decline may reflect the efficacy of science communication strategies that underscore the potential benefits of reef restoration initiatives. However, this positive trajectory experienced disruption in 2024, evidenced by a resurgence of sadness and worry—the most obvious explanation for which is awareness of the mass coral bleaching event that impacted the GBR in the two months immediately prior to execution of the survey in 2024. Other potential explanations include generalised media coverage of reef decline, concerns about the ethical or long-term sustainability of interventions, or broader environmental anxieties tied to intensifying climate impacts. Although our analyses compare waves and include sociodemographic controls, we did not directly model contemporaneous influences (e.g. media coverage, bleaching alerts, policy events). This resurgence of sadness and worry, however, warrants attention because, if persist unchecked, they could undermine public support, corrode hope, or lead to disengagement. Future studies might examine whether this reemergence of negative feelings signals an adaptive emotional response such as vigilance leading to more rigorous policy demands, or a disempowering sense of helplessness.

The normalising of caution over the survey period in our study suggests that respondents are increasingly aware of ecological risks yet not necessarily succumbing to fear. While the proximity of caution to worry suggests a shared undercurrent of concern, its distance from sad and scared suggests caution may support rather than paralyse action. Even emotions seemingly aligned with hope, such as relief, often share meaningful resonances with caution, highlighting how affective and cognitive responses interweave to inform risk perceptions.

Our findings also highlight the complexity of public emotions in response to different novel technological approaches to reef intervention. While protective technologies tend to reduce negative emotions without fostering much optimism, restorative interventions intensify both concerns and hope, reflecting their perceived potential and uncertainty. Adaptive technologies provoke the most polarised reactions, indicating a clear divide in public perception: some view them as risky, while others see them as promising. However, given the small effect size (*η*^2^ = 0.004), these emotional distinctions, while statistically significant, may not translate into substantial shifts in public opinion or policy support. Further research is required to explore these emotional responses in greater depth.

Crucially, these emotional responses do not emerge in isolation but are shaped by underlying social and demographic factors that structure individuals’ vulnerabilities and perceptions of environmental risks. The finding that younger, less educated, or unemployed respondents reported stronger negative emotions aligns with scholarship on environmental injustice, where resource-poor or socially marginalised groups often bear a disproportionate burden of risk and worry (Mohai et al. 2009). Such respondents might perceive themselves as having fewer tools or less power to influence environmental policy, leading to heightened distress or anger. Gender, in contrast, appears in our findings as statistically detectable but substantively negligible. Feminist environmental scholarship has long warned against treating gender as a fixed explanatory factor, instead emphasising its intersection with class, age, labour, and cultural expectations (Ravera et al. 2016). In this sense, the minor gendered differences observed here are better understood as reflective of socially patterned norms about who carries responsibility for care, worry, and attentiveness to harm, rather than as meaningful determinants of how people feel about novel reef interventions.

Consequently, recognising how demographics intersect with emotional responses is paramount for designing inclusive and just governance strategies. By overlooking the emotional dimension of environmental adaptation strategies, we risk perpetuating recognitional injustice among more vulnerable segments of the population (Lau et al. 2021). If the aim is to foster widespread support for reef interventions, we must address the fears and uncertainties of these groups. By acknowledging and validating their emotional experiences, we can develop more genuinely inclusive approaches to environmental adaptation strategies and intervention technologies.

An essential implication of our findings lies in the recognition that emotions are not merely internal, individual experiences. Instead, they are relational forces that intersect with people’s perceived environmental agency. Validating emotions by acknowledging the grief or hope that community members feel can heighten engagement because individuals become more receptive to taking action when they sense their feelings are respected rather than trivialised. Indeed, negative emotions can positively motivate climate-related behaviours, especially when coupled with pathways to action and strengthened coping appraisal (Waters et al. 2024). However, they can also become paralysing if individuals believe their efforts are futile.

Importantly, the interplay of positive and negative emotions is not necessarily contradictory; rather, it can be mutually reinforcing when approached thoughtfully (Waters et al. 2024). Fear, sadness, and anger can serve as wake-up calls, alerting communities to urgent challenges, while hope, pride, and confidence can sustain long-term engagement. Tschakert et al. (2024) argue that positive and negative emotions are not binary opposites but interconnected elements of a broader emotional repertoire necessary for regenerative climate futures. From this vantage, affective ambivalence can be productive: a person might feel anger towards governmental inaction but also hope in the potential of scientific innovation, driving them to advocate for stronger policies while supporting pilot programmes. The richness of these mixed emotions can inspire resilience, creativity, and the willingness to explore novel solutions. At the same time, it must be recognised that negative emotions like fear can also lead to emotional fatigue or denial, while underplaying risk can breed complacency.

Programmes that concentrate solely on ‘the facts’ as they are understood by government and science agencies are insufficient to guide the social and political changes necessary to address global environmental challenges (Nightingale et al. 2022). Integrating emotional insights into policy and governance frameworks is no longer optional, but essential. Emotions inform not only how members of the public react to novel ecosystem interventions but also how stakeholders, from local officials to international NGOs, coordinate their efforts. Interventions that ignore the emotional undercurrents of impacted communities risk fomenting distrust or backlash, especially if they are perceived as technologically intrusive or benefiting an elite few. Conversely, emotionally informed reef governance that actively acknowledges and addresses diverse emotional responses stands a better chance of building broad-based coalitions.

Although situated in the Great Barrier Reef, the contribution of this study lies equally in the way it operationalises emotion as a measurable, longitudinal, and policy-salient dimension of environmental futures. Few empirical works provide a time-sequenced emotional baseline against which emerging intervention pathways can be compared, either within or across geographies. This dataset therefore offers a rare reference point for future comparative studies in other reef systems, coastal regions, or climate-exposed landscapes seeking to understand how collective emotional climates evolve in response to technological propositions. Equally important is the study’s capacity to inform learning between contexts. By revealing which emotions remain durable and which are highly contingent on temporal and contextual shifts, the findings enable policymakers, practitioners and researchers elsewhere to anticipate emotional volatility, calibrate engagement strategies accordingly, and design monitoring frameworks that are sensitive to affective as well as ecological change. In this sense, the work does not propose a universal model of response. Instead, it provides an adaptable empirical scaffold, allowing international communities to situate their own socio-ecological contexts in relation to emerging emotional trajectories under conditions of accelerating environmental transformation.

## Conclusion

Emotions play a pivotal role in shaping how individuals understand and respond to environmental risks and potential solutions (Le et al. 2022; Waters et al. 2024). Far from operating as purely affective noise that hinders rational assessment, emotions can be potent catalysts for collective action or, conversely, deterrents to meaningful policy support. It is striking, in this context, that the strongest emotional response elicited in our research by the prospect of novel interventions to protect, restore and adapt coral reefs in the GBR was hope. It is equally striking that every positive emotional response was stronger than all negative emotional responses with the exception of Caution—a response we have shown to be more reflective of risk awareness than of fear.

In sum, these results show that the provision of information about novel reef interventions has not filled respondents with blind optimism or a myopic attitude towards climate risks. It has served as a cognitive and emotional resource for fostering hope and confidence, rather, in achieving better ecological outcomes than would otherwise be possible in the light of the climate challenge.

Our findings underscore how emotional, technological, and ecological dynamics are interwoven in shaping public support for reef restoration. Far from existing at the margins of rational thought, emotions serve as nuanced, evaluative lenses through which people interpret and engage with novel interventions. By transcending the traditional dichotomy between affect and cognition, we can appreciate the breadth of emotional experiences not as barriers to reasoned decision-making but as integral to it. Indeed, embracing emotional plurality enables more effective policy approaches that value community input, address power imbalances, and amplify the voices of those most at risk. When policymakers and stakeholders see emotions as relational forces co-producing perceptions, behaviours, and collective action, they can harness affective responses to foster sustainable advocacy and bolster adaptive capacities. Validating these shared emotional insights within inclusive governance frameworks catalyses grounded, ethically responsive solutions. Ultimately, recognising emotions as essential forms of evaluative thought will be vital for navigating the uncertainties of reef decline and co-creating resilient, equitable futures for the Great Barrier Reef and beyond.

Future research can build on these findings by delving more deeply into the processes through which emotions translate into tangible policy support or behavioural change (see also Le et al. 2022). While longitudinal survey data offer valuable snapshots of shifting public sentiment, qualitative methods, such as in-depth interviews, focus groups, or ethnographic fieldwork, could illuminate the emotional nuances that do not always emerge in structured questionnaires. Moreover, experimental methods using psycho-physiological techniques may provide more objective measurement of emotions. Using Electrodermal Activity (EDA) sensors can measure heat and emotional activation, while Facial Reading Technology Coding System (FACS) can measure discrete emotions in real time (see Jacobs et al. 2012; Walters et al. 2025). Similarly, investigating the role of communal narratives, media representations, or peer-group norms in shaping emotional responses can further reveal why certain individuals transition from apathy to activism while others remain disengaged. Over time, such work can inform the iterative design of communication strategies, ensuring they remain.

## Supplementary Information

Below is the link to the electronic supplementary material.

Supplementary file1 (PDF 366 KB)

## Data Availability

The data supporting the findings of this study are not publicly available due to ethical restrictions related to participant confidentiality and institutional agreements.
