# Supplementary material for: How do members of the public feel about novel ecosystem interventions? A longitudinal study of emotional responses to restoration and adaptation in the Great Barrier Reef
Source: Ambio. 2026 Jan 9;55(7):1639–53. doi: 10.1007/s13280-025-02329-z (PMC13230461; doi:10.1007/s13280-025-02329-z)
Supplement: Supplementary file 1 — Supplementary file1 (PDF 366 KB) [file 13280_2025_2329_MOESM1_ESM.pdf]

Title:

How do members of the public feel about novel ecosystem interventions? A longitudinal study of emotional responses to restoration and adaptation in the Great Barrier Reef

Word Count:

9700

Authors:

Rana Dadpour (Corresponding author: rana.dadpour@jcu.edu.au , +61 4327 10 100, James Cook University. Address: James Cook University Nguma-Bada campus, 1/14-88 McGregor Rd, Smithfield QLD 4878);

Stewart Lockie (James Cook University Nguma-Bada campus, 1/14-88 McGregor Rd, Smithfield QLD 4878);

Gillian Paxton (James Cook University Nguma-Bada campus, 1/14-88 McGregor Rd, Smithfield QLD 4878);

Brent W. Ritchie (Business School, University of Queensland, Brisbane QLD 4072, Australia)

Appendices

A. Description of novel interventions

We classified the six interventions included in our study into: (1) protective interventions to help protect the Reef from climate extremes; (2) restorative interventions to help support the recovery of degraded reefs; and (3) adaptive interventions to help corals adapt to changing climate conditions. Within each category, we included two specific interventions. Each intervention was introduced to the participant in one paragraph, which included a description of the method used, its aims, and potential benefits and risks. Descriptions of each intervention were developed with scientists and engineers working directly on them to ensure accuracy. Most of the respondents felt that the description of their intervention was neutrally framed, and neutral responses were relatively similar across interventions with no statistical differences found.

For protective interventions, we included marine cloud brightening and fogging (not included in 2018 survey), described to survey respondents as follows:

- One approach is to help cool and shade the waters of the Reef by enhancing the clouds above it to reflect more sunlight and heat. This is known as Marine Cloud Brightening. To achieve this, very small seawater droplets are sprayed into the atmosphere using devices similar to snow making cannons placed on boats, pontoons or permanent structures in the marine environment.

Potential benefits include cooling and shading the reef during stressful conditions that can lead to coral bleaching. Cloud brightening can be used when bleaching conditions are predicted. It can also be used only when it is required (i.e., it can be 'turned off'). Potential risks could include small changes to local weather patterns such as altered rainfall. Further research is required to understand the atmospheric conditions over the Reef, the ability of this technology to be scaled up and the impacts to local weather.

- One approach aims to shade areas of the reef using artificially generated sea mist or fog. This mist reduces the amount of sunlight and heat reaching the sea surface and the coral reefs beneath. Potential benefits include cooling and shading the reef, which prevents damage to coral and other organisms. Fogging could be deployed when coral bleaching conditions (i.e., extreme heat events) are predicted. Fogging could be effective in protecting targeted areas over short critical periods (days to weeks). It can also be used only when it is required (i.e., it can be 'turned off'). Potential risks include over-shading which may slow coral growth. It would be ineffective during strong wind; however, bleaching is most likely to occur during calm conditions.

For restorative interventions, we included rubble stabilization and coral seeding (not included in 2018 survey). These approaches were described to survey respondents as follows:

- One approach aims to help stabilise dead coral rubble so that new corals can grow. Mesh structures or the application of binding agents can be used to

prevent movement of the coral rubble. New coral can then naturally establish themselves and grow on this material. Potential benefits include providing a stable habitat to promote coral settlement and growth. This approach requires minimal human labour to maintain and would work most effectively at a local scale. Potential risks could include changes to natural conditions such as ocean currents, water quality, and impacts on other reef species. Further research is required to understand the effect of this technology. Similar methods are currently used overseas to fix coral reefs damaged by ships.

- One approach – known as coral seeding - involves capturing natural, wild coral spawn/larval slicks in tanks and floating pools and then transporting them for release to help re-seed local or regional high-priority reefs. Potential benefits include speeding up the return of coral cover to a disturbed or damaged reef by increasing the number of available coral babies (larvae) that can settle and grow into new corals. Harvesting wild coral larval slicks is likely to have a minimal negative impact on Reef ecology, as the mortality rate of the larvae in a slick is typically high. Potential risks include introduced and relocated corals impacting the local coral population ecosystem. More research is needed to develop effective and efficient collection and transfer and delivery techniques.

For adaptive interventions, we included enhancing heat resistance – natural breeding and enhancing heat resistance – genetic engineering. These approaches were described to survey respondents as follows:

- One approach aims to enhance the heat resistance of corals through the selective breeding and spread of naturally more heat resistant corals. This can help corals become more resilient to ocean warming and improve the overall health of coral reefs. Potential benefits include helping corals to become more tolerant to marine heatwaves and environmental change. Potential risks could include possible changes in local coral structures and species interactions. Further research is required to understand the effect of this technology. This approach may involve breeding corals in land-based nurseries, an approach that is already used in aquaculture.
- One potential approach could enhance the heat resistance of corals using genetic engineering. Genetic engineering includes methods that allow very precise changes to be made to the genetic material of corals, or algae that lives in corals, to increase their ability to cope with heat stress. Potential benefits include helping corals to resist, repair and recover from bleaching events by improving their tolerance to heat stress. Potential risks could include possible disruptions to the ecosystem and spread of modified algae to other organisms. This technology is not yet sufficiently developed to be applied to corals. Further research is required to understand the effect of this technology. Similar methods have been used to genetically modify food crops including maize and canola.

B. Descriptive Statistics

Descriptive Statistics

		Statistic	Std. Error	Bootstrap[a] Bias	Std. Error	95% Confidence Interval Lower	Upper
How do you feel when you consider the type of approach/technology outlined earlier? Cautious	N	8459		0	0	8459	8459
	Minimum	1					
	Maximum	7					
	Mean	4.30		.00	.02	4.28	4.32
	Std. Deviation	1.500		-.001	.011	1.480	1.520
	Skewness	-.125	.027	.001	.015	-.153	-.093
	Kurtosis	-.109	.053	.001	.033	-.168	-.043
How do you feel when you consider the type of approach/technology outlined earlier? Worried	N	8459		0	0	8459	8459
	Minimum	1					
	Maximum	7					
	Mean	3.53		.00	.02	3.49	3.56
	Std. Deviation	1.625		.000	.011	1.604	1.646
	Skewness	.175	.027	.000	.015	.148	.207
	Kurtosis	-.541	.053	.000	.024	-.588	-.490
How do you feel when you consider the type of approach/technology outlined earlier? Sadness	N	8459		0	0	8459	8459
	Minimum	1.00					
	Maximum	7.00					
	Mean	3.0437		.0004	.0182	3.0067	3.0805
	Std. Deviation	1.74303		.00021	.01122	1.72023	1.76421
	Skewness	.493	.027	.000	.015	.461	.521
	Kurtosis	-.625	.053	.001	.024	-.673	-.578
How do you feel when you consider the type of approach/technology outlined earlier? Powerless	N	8459		0	0	8459	8459
	Minimum	1.00					
	Maximum	7.00					
	Mean	3.6094		-.0003	.0183	3.5704	3.6464
	Std. Deviation	1.69879		.00027	.01113	1.67867	1.72428
	Skewness	.329	.027	.000	.014	.303	.356
	Kurtosis	-.701	.053	.001	.022	-.741	-.658
How do you feel when you consider the type of approach/technology outlined earlier? Scared	N	8459		0	0	8459	8459
	Minimum	1.00					
	Maximum	7.00					
	Mean	3.0161		.0000	.0184	2.9772	3.0611
	Std. Deviation	1.68565		-.00006	.01066	1.66168	1.70667
	Skewness	.479	.027	.000	.015	.447	.512
	Kurtosis	-.563	.053	.001	.025	-.610	-.512
How do you feel when you consider the type of approach/technology outlined earlier? Proud	N	8459		0	0	8459	8459
	Minimum	1.00					
	Maximum	7.00					
	Mean	4.0683		-.0000	.0173	4.0518	4.1163
	Std. Deviation	1.62025		-.00060	.01059	1.60407	1.62271
	Skewness	-.218	.027	.000	.013	-.244	-.188
	Kurtosis	.461	.053	.000	.028	.459	.358
How do you feel when you consider the type of approach/technology outlined earlier? Hopeful	N	8459		0	0	8459	8459
	Minimum	1.00					
	Maximum	7.00					
	Mean	4.0587		.0007	.0181	4.0665	4.9018
	Std. Deviation	1.76683		-.00046	.01157	1.74268	1.78746
	Skewness	-.044	.027	.000	.018	-.063	-.006
	Kurtosis	.169	.053	.001	.036	.177	.367
How do you feel when you consider the type of approach/technology outlined earlier? Happy	N	8459		0	0	8459	8459
	Minimum	1.00					
	Maximum	7.00					
	Mean	4.2817		.0002	.0167	4.2485	4.3157
	Std. Deviation	1.51542		-.00060	.01125	1.49218	1.53712
	Skewness	-.266	.027	.000	.015	-.325	-.267
	Kurtosis	-.063	.053	.002	.032	-.148	-.027
How do you feel when you consider the type of approach/technology outlined earlier? Confident	N	8459		0	0	8459	8459
	Minimum	1.00					
	Maximum	7.00					
	Mean	4.2705		.0002	.0162	4.2353	4.3048
	Std. Deviation	1.47675		-.00006	.01140	1.45432	1.49766
	Skewness	-.253	.027	.000	.015	-.281	-.217
	Kurtosis	-.071	.053	.002	.033	-.135	-.003
How do you feel when you consider the type of approach/technology outlined earlier? Relieved	N	8459		0	0	8459	8459
	Minimum	1.00					
	Maximum	7.00					
	Mean	4.1355		.0007	.0171	4.1024	4.1650
	Std. Deviation	1.54635		-.00060	.01033	1.52532	1.56625
	Skewness	-.203	.027	.000	.014	-.237	-.161
	Kurtosis	-.337	.053	.002	.033	-.383	-.464
Valid N (listwise)	N	8459		0	0	8459	8459

a. Unless otherwise noted, bootstrap results are based on 1000 bootstrap samples

C. Clusters of Emotions

Spearman's correlation matrix

Correlations

			How do you feel when you consider the type of approach/technology outlined earlier? Cautious	How do you feel when you consider the type of approach/technology outlined earlier? Worried	How do you feel when you consider the type of approach/technology outlined earlier? Sadness	How do you feel when you consider the type of approach/technology outlined earlier? Powerless	How do you feel when you consider the type of approach/technology outlined earlier? Scared	How do you feel when you consider the type of approach/technology outlined earlier? Proud	How do you feel when you consider the type of approach/technology outlined earlier? Hopeful	How do you feel when you consider the type of approach/technology outlined earlier? Happy	How do you feel when you consider the type of approach/technology outlined earlier? Confident	How do you feel when you consider the type of approach/technology outlined earlier? Relieved
Spearman's rho	How do you feel when you consider the type of approach/technology outlined earlier? Cautious	Correlation Coefficient	1.000	.479**	.310**	.321**	.361**	.035**	.356**	.004	.003	.037**
		Sig. (2-tailed)	.	<.001	<.001	<.001	<.001	.001	<.001	.701	.816	<.001
		N	8459	8459	8459	8459	8459	8459	8459	8459	8459	8459
	How do you feel when you consider the type of approach/technology outlined earlier? Worried	Correlation Coefficient	.479**	1.000	.633**	.516**	.646**	-.030**	-.121**	-.128**	-.133**	-.057**
		Sig. (2-tailed)	<.001	.	<.001	<.001	<.001	.005	<.001	<.001	<.001	<.001
		N	8459	8459	8459	8459	8459	8459	8459	8459	8459	8459
	How do you feel when you consider the type of approach/technology outlined earlier? Sadness	Correlation Coefficient	.310**	.633**	1.000	.570**	.678**	.004	-.141**	-.101**	-.087**	-.011
		Sig. (2-tailed)	<.001	<.001	.	<.001	<.001	.711	<.001	<.001	<.001	.326
		N	8459	8459	8459	8459	8459	8459	8459	8459	8459	8459
	How do you feel when you consider the type of approach/technology outlined earlier? Powerless	Correlation Coefficient	.321**	.516**	.570**	1.000	.669**	-.023*	-.116**	-.101**	-.123**	-.051**
		Sig. (2-tailed)	<.001	<.001	<.001		<.001	.032	<.001	<.001	<.001	<.001
		N	8459	8459	8459	8459	8459	8459	8459	8459	8459	8459
	How do you feel when you consider the type of approach/technology outlined earlier? Scared	Correlation Coefficient	.361**	.646**	.678**	.669**	1.000	.058**	-.115**	-.052**	-.067**	.032**
		Sig. (2-tailed)	<.001	<.001	<.001	<.001		<.001	<.001	<.001	<.001	.003
		N	8459	8459	8459	8459	8459	8459	8459	8459	8459	8459
	How do you feel when you consider the type of approach/technology outlined earlier? Proud	Correlation Coefficient	.035**	-.030**	.004	-.023*	.058**	1.000	.563**	.580**	.650**	.649**
		Sig. (2-tailed)	.001	.005	.711	.032	<.001	.	<.001	<.001	<.001	<.001
		N	8459	8459	8459	8459	8459	8459	8459	8459	8459	8459
	How do you feel when you consider the type of approach/technology outlined earlier? Hopeful	Correlation Coefficient	.356**	.121**	.141**	.116**	.115**	.563**	1.000	.535**	.521**	.583**
		Sig. (2-tailed)	<.001	<.001	<.001	<.001	<.001	<.001	.	<.001	<.001	<.001
		N	8459	8459	8459	8459	8459	8459	8459	8459	8459	8459
	How do you feel when you consider the type of approach/technology outlined earlier? Happy	Correlation Coefficient	.004	-.128**	-.101**	-.101**	-.052**	.580**	.536**	1.000	.719**	.695**
		Sig. (2-tailed)	.701	<.001	<.001	<.001	<.001	<.001	<.001	.	<.001	<.001
		N	8459	8459	8459	8459	8459	8459	8459	8459	8459	8459
	How do you feel when you consider the type of approach/technology outlined earlier? Confident	Correlation Coefficient	.003	.133**	.087**	.123**	.067**	.650**	.521**	.715**	1.000	.688**
		Sig. (2-tailed)	.816	<.001	<.001	<.001	<.001	<.001	<.001	<.001	.	<.001
		N	8459	8459	8459	8459	8459	8459	8459	8459	8459	8459
	How do you feel when you consider the type of approach/technology outlined earlier? Relieved	Correlation Coefficient	.037**	-.057**	-.011	-.051**	.032**	.649**	.583**	.695**	.688**	1.000
		Sig. (2-tailed)	<.001	<.001	.326	<.001	.003	<.001	<.001	<.001	<.001	.
		N	8459	8459	8459	8459	8459	8459	8459	8459	8459	8459

**. Correlation is significant at the 0.01 level (2-tailed).

*. Correlation is significant at the 0.05 level (2-tailed).

Proximity Matrix

Proximity Matrix

Case	Matrix File Input									
	How do you feel when you consider the type of approach/technology outlined earlier?. Cautious	How do you feel when you consider the type of approach/technology outlined earlier?. Worried	How do you feel when you consider the type of approach/technology outlined earlier?. Sadness	How do you feel when you consider the type of approach/technology outlined earlier?. Powerless	How do you feel when you consider the type of approach/technology outlined earlier?. Scared	How do you feel when you consider the type of approach/technology outlined earlier?. Proud	How do you feel when you consider the type of approach/technology outlined earlier?. Hopeful	How do you feel when you consider the type of approach/technology outlined earlier?. Happy	How do you feel when you consider the type of approach/technology outlined earlier?. Confident	How do you feel when you consider the type of approach/technology outlined earlier?. Relieved
How do you feel when you consider the type of approach/technology outlined earlier?. Cautious	.000	25558.000	43022.000	35096.000	40194.000	40707.000	38937.000	38844.000	38003.000	38429.000
How do you feel when you consider the type of approach/technology outlined earlier?. Worried	26558.000	.000	20480.000	23106.000	18298.000	48691.000	60919.000	51528.000	50633.000	47705.000
How do you feel when you consider the type of approach/technology outlined earlier?. Sadness	43022.000	20480.000	.000	24144.000	16432.000	56561.000	77763.000	61920.000	59787.000	55949.000
How do you feel when you consider the type of approach/technology outlined earlier?. Powerless	35096.000	23106.000	24144.000	.000	24034.000	52617.000	64977.000	54772.000	54633.000	51975.000
How do you feel when you consider the type of approach/technology outlined earlier?. Scared	40194.000	18298.000	16432.000	24034.000	.000	52909.000	75645.000	58466.000	57737.000	52835.000
How do you feel when you consider the type of approach/technology outlined earlier?. Proud	40707.000	48691.000	56561.000	52617.000	52909.000	.000	21910.000	13199.000	14230.000	14196.000
How do you feel when you consider the type of approach/technology outlined earlier?. Hopeful	38937.000	60919.000	77763.000	64977.000	75645.000	21910.000	.000	16049.000	16040.000	19932.000
How do you feel when you consider the type of approach/technology outlined earlier?. Happy	38844.000	51528.000	61920.000	54772.000	58466.000	13199.000	16049.000	.000	9951.000	11335.000
How do you feel when you consider the type of approach/technology outlined earlier?. Confident	38003.000	50633.000	59787.000	54633.000	57737.000	14230.000	16040.000	9951.000	.000	11360.000
How do you feel when you consider the type of approach/technology outlined earlier?. Relieved	38429.000	47705.000	55949.000	51975.000	52835.000	14196.000	19932.000	11335.000	11360.000	.000
